# Compared effectiveness of sodium zirconium cyclosilicate and calcium polystyrene sulfonate on hyperkalemia in patients with chronic kidney disease

**DOI:** 10.3389/fmed.2023.1137981

**Published:** 2023-03-06

**Authors:** Takashin Nakayama, Shintaro Yamaguchi, Kaori Hayashi, Kiyotaka Uchiyama, Takaya Tajima, Tatsuhiko Azegami, Kohkichi Morimoto, Tadashi Yoshida, Jun Yoshino, Toshiaki Monkawa, Takeshi Kanda, Hiroshi Itoh

**Affiliations:** ^1^Division of Endocrinology, Metabolism and Nephrology, Department of Internal Medicine, Keio University School of Medicine, Tokyo, Japan; ^2^Keio University Health Center, Yokohama, Japan; ^3^Apheresis and Dialysis Center, Keio University School of Medicine, Tokyo, Japan; ^4^Medical Education Center, Keio University School of Medicine, Tokyo, Japan

**Keywords:** chronic kidney disease, electrolytes, hyperkalemia, calcium polystyrene sulfonate, sodium zirconium cyclosilicate, metabolic acidosis, serum sodium minus chloride level

## Abstract

Hyperkalemia is a well-recognized electrolyte abnormality in patients with chronic kidney disease (CKD). Potassium binders are often used to prevent and treat hyperkalemia. However, few studies have evaluated the difference in serum potassium (K^+^) level-lowering effect during the post-acute phase between the novel potassium binder, sodium zirconium cyclosilicate (ZSC), and conventional agents. This retrospective study included patients who received potassium binders (either ZSC or calcium polystyrene sulfonate [CPS]) in our hospital between May 2020 and July 2022. The patients were divided into the ZSC and CPS groups. After propensity score matching, we compared changes from baseline to the first follow-up point, at least 4 weeks after initiating potassium binders, in electrolytes including K^+^ level between the two groups. Of the 132 patients, ZSC and CPS were administered in 48 and 84 patients, respectively. After matching, 38 patients were allocated to each group. The ZSC group showed greater reduction in K^+^ levels than did the CPS group (*P* < 0.05). Moreover, a significant increase in serum sodium minus chloride levels, a surrogate marker for metabolic acidosis, was observed in the ZSC group (*P* < 0.05). Our results demonstrated that ZSC could potentially improve hyperkalemia and metabolic acidosis in patients with CKD.

## Introduction

Hyperkalemia is a common complication in patients with chronic kidney disease (CKD) and heart failure, particularly with the use of renin-angiotensin system (RAS) inhibitors, which are the cornerstone of treatment of these diseases (1–4). This electrolyte abnormality is strongly associated with increased mortality and healthcare costs in the short and long term. Therefore, optimal management of serum potassium (K^+^) levels, which reduces clinical complications and economic burden, is crucially important (5–9).

Dietary potassium restriction has been recommended for preventing and treating hyperkalemia in patients with CKD (10). However, adherence to renal diet restrictions is likely to be inadequate even in patients with sufficient knowledge (11). As such, use of potassium binders, which bind potassium in the gastrointestinal tract to increase its fecal elimination, is also a therapeutic option for hyperkalemia. Although organic polymer resins such as calcium polystyrene sulfonate (CPS) and sodium polystyrene sulfonate have been conventionally used, their efficacy and safety have been concerned. Utilizing these resins, gastrointestinal toxicity is common as treatment-related adverse events. Furthermore, recurrent hyperkalemia episodes over a short period of time are frequent (12, 13). Therefore, clinicians often make decision with compromise to discontinue or down-titrate RAS inhibitors, which could increase the risk of death and cardiovascular events (7, 8, 14, 15).

Sodium zirconium cyclosilicate (ZSC), a novel potassium binder, was approved for use in Japan on March 26, 2020. Compared with conventional nonspecific organic polymer resins, this inorganic cation exchange compound has a capacity for more selectively and efficiently capturing monovalent cations, particularly excess potassium and ammonium (NH4^+^) (16, 17). Additionally, ZSC does not absorb water, which may reduce the risk of potassium binder-related constipation. Importantly, unlike conventional potassium binders requiring frequent medications, ZSC is essentially administered as a once-daily dose, leading to improved adherence to medication (18). Although these profiles imply that ZSC could be an attractive therapeutic agent, few studies have evaluated its efficacy and safety in real–world practice settings.

Therefore, we conducted this retrospective study to compare the effect of ZSC and conventional potassium binders on changes in electrolytes and kidney function in patients with CKD.

## Materials and methods

### Study participants

Considering the prescription status at our hospital, we defined CPS as a conventional potassium binder. All patients aged ≥18 years who have newly started ZSC or CPS in our hospital between May 2020 and July 2022 were included in the study. CPS comprised ARGAMATE 20% JELLY 25 g (Astellas Pharma Inc.), CALCIUM POLYSTYRENE SULFONATE 20% Oral Jelly “SANWA” (Sanwa Kagaku Kenkyusho Co., Ltd.) or Kalimate Oral Solution 20% (Kowa Pharmaceuticals Co., Ltd.). ZSC only included Lokelma 5 g or 10 g powder for oral suspension (AstraZeneca, plc.). Precisely assessing the effect of potassium binders in patients with acute hyperkalemia may be difficult because insulin-glucose infusion or beta-2 adrenergic agonist inhalation is often involved. Therefore, we excluded patients who had been treated for <4 weeks to evaluate the effects of potassium binders in the post-acute phase. The other exclusion criteria were: estimated glomerular filtration rate (eGFR) > 60 mL/min/1.73 m^2^; patients on dialysis; and missing data on K^+^ level before or after administration. This was a single-center retrospective cohort study. All protocols were reviewed and approved by the Keio University School of Medicine Ethics Committee (approval no.: 20221131). Informed consent was obtained using the opt-out method available on the website.

### Clinical parameters

We collected the following demographic and anthropometric data from medical records: age, sex, disposition status (inpatient or outpatient), comorbid conditions, and primary cause of CKD. Data of medications associated with serum potassium level (RAS inhibitors, mineralocorticoid receptor [MR] blockers, loop diuretics, thiazide diuretics, beta blockers, or sodium bicarbonate), body weight (BW) (kg), and blood pressure (mmHg) were also collected. The comorbidity score was assessed using the Charlson Comorbidity Index (CCI). Additionally, we collected biochemical data on potassium binder initiation and the first follow-up, 4 weeks after administration. These biochemical data included serum levels of albumin (g/dl), urea nitrogen (mg/dl), creatinine (mg/dl), sodium (mEq/l), chloride (mEq/l), potassium (mEq/l), calcium (mg/dl), and phosphorus (mg/dl). Additionally, eGFR (ml/min/1.73 m^2^) was calculated using the 3-variable Japanese equation: eGFR = 194 × serum Creatine^−1.094^ × Age^−0.287^ (×0.739 if woman) (19). The corrected calcium level was obtained for the lower range of albumin using Payne's formula: corrected total calcium = total calcium + (4.0—albumin) (20). Since data of serum bicarbonate level was unavailable, sodium minus chloride level was used as a surrogate marker for evaluating metabolic acidosis (21).

Daily drug cost for CPS and ZSC was calculated using the following values: 68.4 JPY/pack for ARGAMATE 20% JELLY 25 g, 61.4 JPY/pack for CALCIUM POLYSTYRENE SULFONATE 20% Oral Jelly “SANWA”, 66.1 JPY/pack for Kalimate Oral Solution 20%, 1,069.3 JPY/pack for Lokelma 5 g and 1,567.0 JPY/pack for Lokelma 10 g (22, 23).

To evaluate tolerability, we reviewed electronic medical records to determine whether the patients continued each potassium binder beyond the follow-up point. Additionally, if discontinued, we evaluated the reasons for cessation.

### Statistical analyses

Continuous variables are expressed as medians (25th to 75th percentiles). Binary variables are expressed as percentages. Differences between the patients receiving ZSC and those administered CPS in normally and non-normally distributed continuous variables (assessed using the Kolmogorov-Smirnov test) and binary variables were evaluated using the unpaired Student's *t*-test, Mann–Whitney U test, and Fisher's exact test, respectively.

Propensity score matching was employed to adjust for confounding variables and reduce treatment selection bias. We included the following clinically potential predictors of changes in K^+^ levels and selection of potassium binder type as independent variables: age, sex, BW, CCI, baseline eGFR, baseline K^+^ level, and use of RAS inhibitors. Propensity scores were calculated using a logistic regression model based on these variables. Subsequently, the ZSC and CPS groups were matched 1:1 on the logit of the propensity score using calipers with 0.2 standard deviation of the logit of the propensity score (24).

In a sensitivity analysis, a linear mixed effect model analysis was performed to evaluate the K^+^-lowering effect of ZSC. Fixed effects were time (baseline and follow-up) and time × potassium binder type besides variables that were selected for the above-mentioned propensity score, and random effect was participant number. Moreover, changes in sodium minus chloride levels, corrected calcium levels, phosphorus levels, urea nitrogen levels, creatinine levels, and eGFR were evaluated using a linear mixed effect model with each baseline value added to the fixed effects. Outcomes of these models are expressed as estimated marginal means (95% confidence interval).

All statistical analyses were performed using SPSS version 27 (IBM Inc., Armonk, NY, USA) and EZR, which is a graphical user interface for R (The R Foundation for Statistical Computing) (25). All *P*-values were two-sided, and those < 0.05 indicated statistically significance.

## Results

### Patients' characteristics

**Figure 1:**
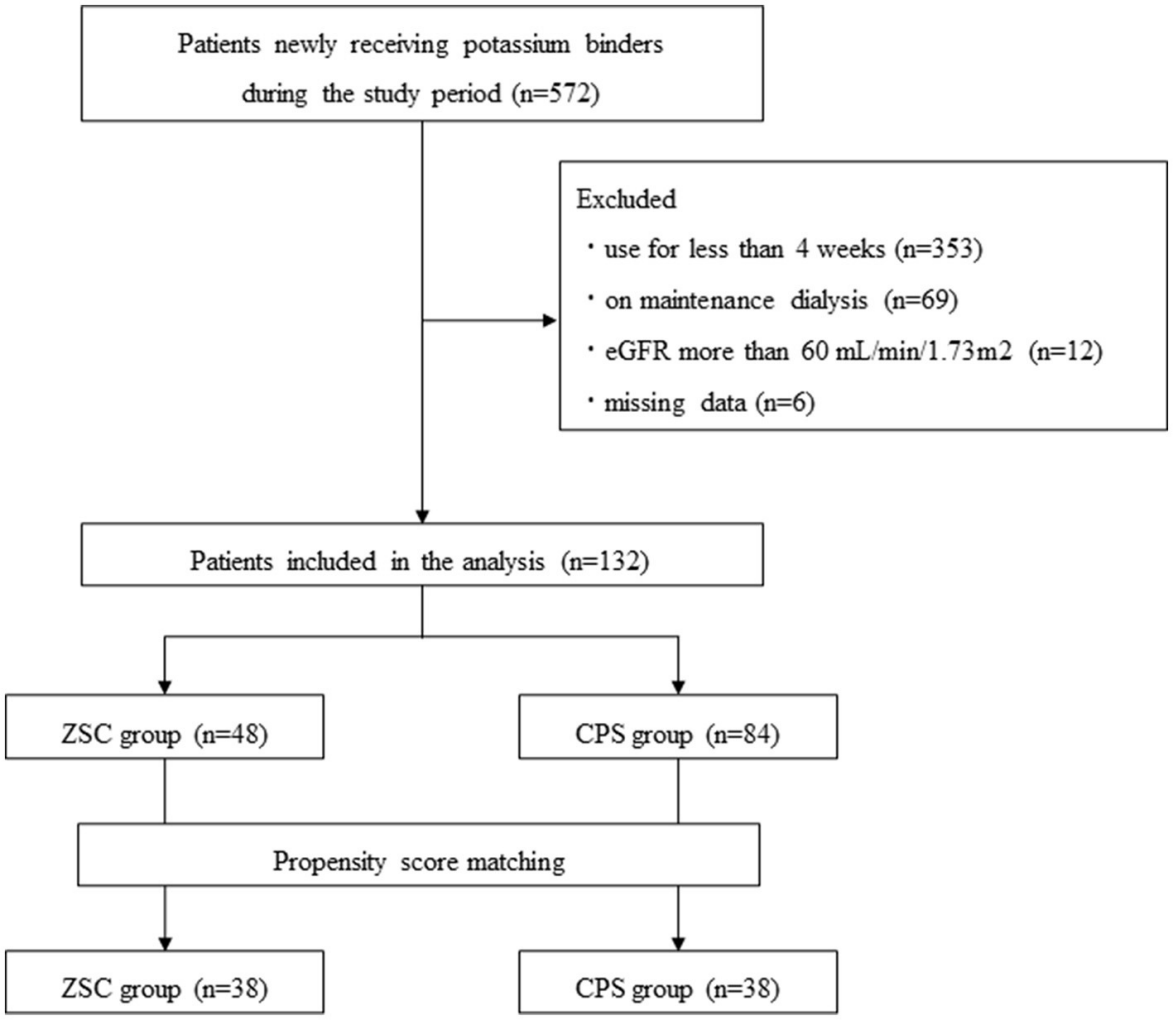
Flow chart of the study enrollment.

Among a total of 572 patients who newly received potassium binders (ZSC or CPS) during the study period, 440 patients were excluded. Those excluded were patients using ZSC or CPS for <4 weeks (*n* = 353), on dialysis (*n* = 69), with eGFR > 60 mL/min/1.73 m^2^ (*n* = 12), and missing values of pre and/or post K^+^ levels (*n* = 6) (Figure 1). Consequently, 132 patients administered ZSC (*n* = 48) and CPS (*n* = 84) were included in the study: 38 patients were matched in both groups. Table 1 summarizes the baseline characteristics of the two groups, categorized according to the type of potassium binder used. Before propensity score matching, in the ZSC and CPS groups, the median age was 75 and 79 years and the female proportion was 18.8 and 33.3%, respectively. The follow-up period did not significantly differ between the two groups (6 vs. 5 weeks, *P* = 0.52). Regarding medication-related burden, the number of doses per day was significantly lower in the ZSC group than in the CPS group (1 vs. 2, *P* < 0.01), whereas the ZSC group had a significantly higher daily medication cost (1,069 vs. 132 JPY, *P* < 0.01). The ZSC group tended to be more likely to receive RAS inhibitors than did the CPS group (*P* = 0.07). However, no significant difference was observed in the frequency of RAS inhibitor use after matching between the two groups.

**Table 1:** Baseline characteristics according to the type of potassium binders.

**Variables**	**All patients**			**Propensity-matched patients**		
	**ZSC group (*****n** =* **48)**	**CPS group (*****n** =* **84)**	***P*** **value**	**ZSC group (*****n** =* **38)**	**CPS group (*****n** =* **38)**	***P*** **value**
Age, years	75 (69–84)	79 (71–84)	0.63	76 (70–84)	80 (71–84)	0.73
Sex (female)	9 (18.8)	28 (33.3)	0.11	8 (21.1)	9 (23.7)	1.00
Disposition (Inpatient)	9 (18.8)	13 (15.5)	0.64	8 (21.1)	7 (18.4)	1.00
Follow-up period (week)	6 (4–7)	5 (4–7)	0.52	6 (4–7)	5 (4–7)	0.62
**Dosage regimen**						
Dosage per day (g)	5 (5)	10 (5–15)	< 0.01	5 (5)	10 (5–15)	< 0.01
Number of doses per day	1 (1)	2 (1–3)	< 0.01	1 (1)	2 (1–3)	< 0.01
Cost per day (JPY)	1,069 (1,069–1,069)	132 (66–188)	< 0.01	1,069 (1,069–1,069)	132 (66–195)	< 0.01
Body weight (kg)	60.4 (52.6–66.0)	59.9 (48.7–68.3)	0.98	58.1 (50.6–66.0)	57.6 (47.9–66.3)	0.60
Mean BP (mmHg)	92 (81–103)	91 (81–101)	0.51	91 (81–102)	88 (82–101)	0.37
**Comorbidities**						
Diabetes	21 (43.8)	32 (38.1)	0.58	15 (39.5)	13 (34.2)	0.81
Coronary artery disease	14 (29.2)	18 (21.4)	0.40	10 (26.3)	10 (26.3)	1.00
Congestive heart failure	19 (39.6)	39 (46.4)	0.47	16 (42.1)	22 (57.9)	0.25
Cerebrovascular disease	7 (14.6)	16 (19.0)	0.64	5 (13.2)	7 (18.4)	0.75
Malignancy	18 (37.5)	27 (32.1)	0.57	16 (42.1)	13 (34.2)	0.64
CCI	4 (2–5)	4 (3–5)	0.93	4 (3–5)	4 (2–5)	0.80
**Underlying disease**						
Diabetic kidney disease	14 (29.2)	29 (34.5)	0.87	9 (23.7)	12 (31.6)	0.40
Nephrosclerosis	15 (31.2)	21 (25.0)		14 (36.8)	13 (34.2)	
Glomerulonephritis	4 (8.3)	8 (9.5)		2 (5.3)	5 (13.2)	
Others	15 (31.2)	26 (31.0)		13 (34.2)	8 (21.1)	
**Medications**						
RAS inhibitors	29 (60.4)	36 (42.9)	0.07	20 (52.6)	19 (50.0)	1.00
MR blockers	7 (14.6)	8 (9.5)	0.40	4 (10.5)	3 (7.9)	1.00
Loop diuretics	7 (14.6)	21 (25.0)	0.19	6 (15.8)	11 (28.9)	0.27
Thiazide	3 (6.2)	7 (8.3)	0.75	3 (7.9)	2 (5.3)	1.00
Beta blockers	15 (31.2)	24 (28.6)	0.84	12 (31.6)	14 (36.8)	0.81
Sodium bicarbonate	7 (14.6)	10 (11.9)	0.79	6 (15.8)	8 (21.1)	0.77

ZSC, sodium zirconium cyclosilicate; CPS, calcium polystyrene sulfonate; BP, blood pressure; CCI, charlson comorbidity index; RAS, renin-angiotensin system; MR, mineralocorticoid receptor.

### Efficacy in lowering K^+^ level

**Figure 2:**
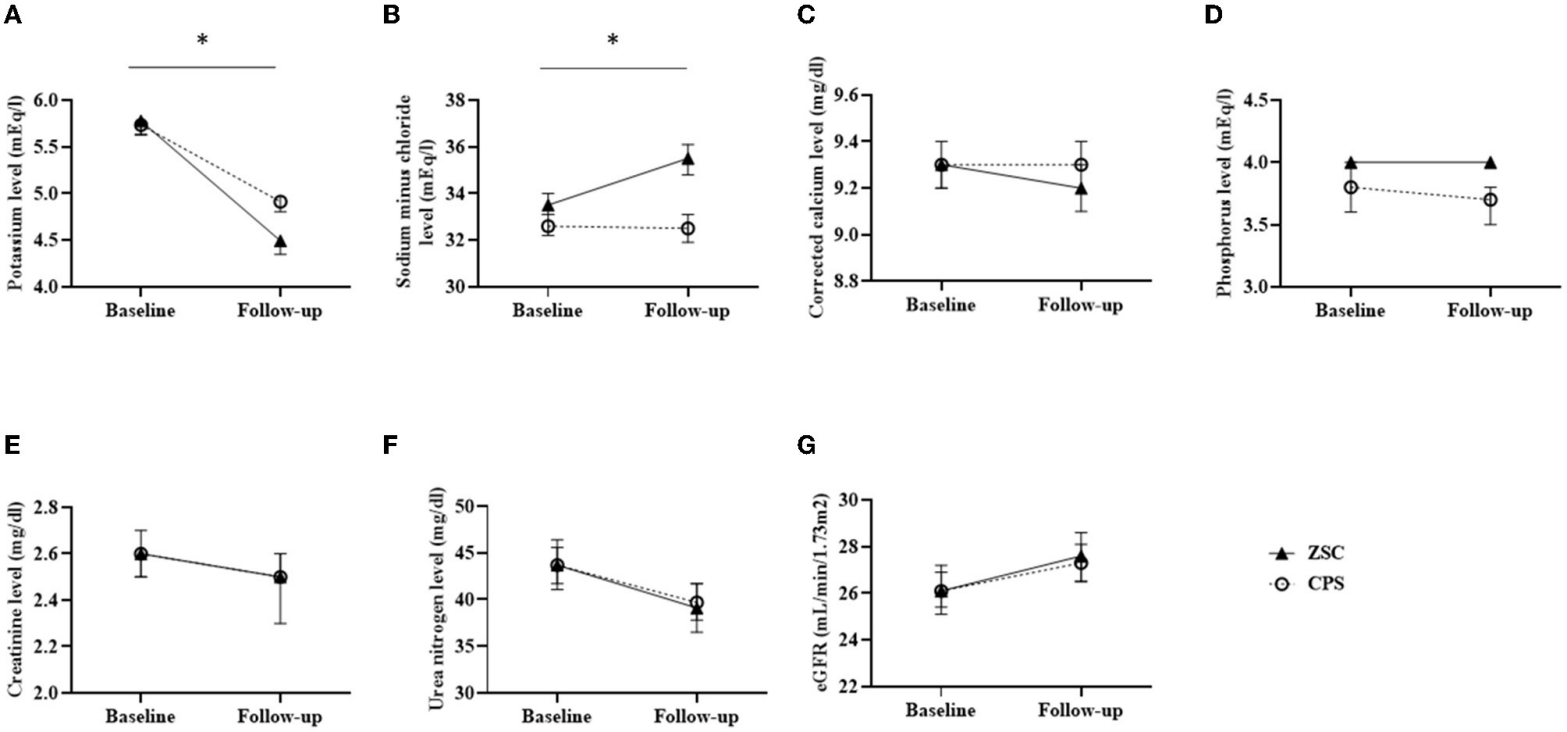
Estimated marginal means in biochemical data changes assessed by linear mixed effect models. **(A)** Potassium level (mEq/l) **(B)** Sodium minus chloride level (mEq/l) **(C)** Corrected calcium level (mg/dl) **(D)** Phosphorus (mg/dl) **(E)** Creatinine level (mg/dl) **(F)** Urea nitrogen level (mg/dl) **(G)** eGFR (mL/min/1.73m^2^). Error bars representing 95% confidence interval at each point. *P < 0.01.

The K^+^ levels are listed in Table 2. Compared with the CPS group, the ZSC group tended to have high baseline K^+^ level (5.9 vs. 5.7 mEq/l, *P* = 0.07). However, propensity score matching eliminated that significant difference (5.8 vs. 5.8 mEq/l, *P* = 0.76). After matching, the follow-up K^+^ level was lower in the ZSC group than that in the CPS group (4.5 vs. 5.0 mEq/l, *P* < 0.05). Moreover, the ZSC group showed a greater reduction in K^+^ levels (−1.2 vs. −0.8 mEq/l, *P* < 0.05) compared with the CPS group. Linear mixed effect model analysis revealed that K^+^ levels with estimated marginal mean in the ZSC group declined from 5.8 at baseline to 4.5 mEq/l at follow-up, whereas it declined from 5.7 to 4.9 mEq/l in the CPS group (Figure 2A; Supplementary Table S1), indicating a mean difference of −0.5 mEq/l between the two group (*P* < 0.01).

**Table 2:** Laboratory data according to the type of potassium binders.

**Variables**	**All patients**			**Propensity-matched patients**		
	**ZSC group (*****n** =* **48)**	**CPS group (*****n** =* **84)**	***P*** **value**	**ZSC group (*****n** =* **38)**	**CPS group (*****n** =* **38)**	***P*** **value**
**Potassium (mEq/L)**						
Baseline ^a^	5.9 (5.6 to 6.1)	5.7 (5.5 to 6.1)	0.07	5.8 (5.5 to 6.1)	5.8 (5.4 to 6.1)	0.76
Follow–up ^a^	4.6 (4.2 to 4.9)	4.7 (4.4 to 5.4)	< 0.05	4.5 (4.1 to 4.9)	5.0 (4.6 to 5.4)	< 0.05
Difference ^a^	−1.3 (−1.5 to −0.8)	−0.8 (−1.3 to −0.5)	< 0.01	−1.2 (−1.4 to −0.7)	−0.8 (−1.3 to −0.3)	< 0.05
**Sodium minus chloride (mEq/L)**						
Baseline ^a^	32.4 (29.3 to 34.5)	32.5 (30.9 to 35.0)	0.11	32.3 (29.2 to 34.5)	31.9 (30.3 to 34.6)	0.90
Follow–up ^a^	34.8 (32.8 to 37.0)	33.5 (31.8 to 35.7)	0.05	35.2 (32.2 to 37.6)	33.1 (31.5 to 34.9)	< 0.05
Difference ^b^	1.9 (0.5 to 4.5)	0.8 (−0.6 to 2.5)	< 0.01	1.9 (0.6 to 4.4)	1.0 (−0.5 to 2.4)	< 0.05
**Corrected calcium (mg/dL)**						
Baseline ^a^	9.3 (9.0 to 9.6)	9.3 (9.0 to 9.6)	0.99	9.4 (9.0 to 9.6)	9.2 (8.9 to 9.6)	0.52
Follow–up ^a^	9.2 (9.0 to 9.5)	9.3 (9.0 to 9.7)	0.43	9.2 (9.0 to 9.5)	9.2 (9.0 to 9.7)	0.52
Difference ^a^	0.0 (−0.3 to 0.1)	0.0 (−0.2 to 0.3)	0.24	0.0 (−0.3 to 0.1)	0.1 (−0.1 to 0.3)	0.05
**Phosphorus (mg/dL)**						
Baseline ^a^	3.9 (3.7 to 4.6)	3.8 (3.4 to 4.5)	0.60	3.9 (3.7 to 4.5)	3.8 (3.3 to 4.3)	0.31
Follow–up ^a^	3.7 (3.3 to 4.1)	3.6 (3.1 to 4.0)	0.38	3.7 (3.4 to 4.1)	3.7 (3.2 to 4.1)	0.19
Difference ^a^	−0.2 (−0.6 to 0.1)	−0.3 (−0.6 to 0.1)	0.53	−0.2 (−0.6 to 0.1)	−0.3 (−0.4 to 0.2)	0.62
**Creatinine (mg/dL)**						
Baseline ^b^	2.1 (1.7 to 3.3)	2.0 (1.2 to 3.2)	0.19	2.4 (1.7 to 3.5)	2.3 (1.3 to 3.2)	0.47
Follow–up ^b^	2.0 (1.6 to 3.1)	1.8 (1.2 to 3.2)	0.28	2.3 (1.7 to 3.4)	2.1 (1.3 to 3.2)	0.54
Difference ^b^	−0.1 (−0.3 to 0.0)	0.0 (−0.2 to 0.1)	0.06	0.0 (−0.2 to 0.1)	0.0 (−0.1 to 0.1)	0.24
**Urea nitrogen (mg/dL)**						
Baseline ^a^	44.1 (34.2 to 55.2)	38.9 (27.9 to 54.0)	0.27	44.2 (34.6 to 57.0)	40.7 (29.9 to 55.6)	0.53
Follow–up ^a^	35.7 (30.1 to 45.7)	36.4 (24.9 to 48.5)	0.76	39.0 (31.1 to 48.7)	40.4 (25.7 to 51.9)	0.84
Difference ^a^	−7.3 (−14.8 to 1.2)	−2.5 (−8.5 to 2.0)	0.07	−5.1 (−13.7 to 2.2)	−1.9 (−7.7 to 3.1)	0.22
**eGFR (mL/min/1.73m2)**						
Baseline ^a^	22 (14 to 32)	25 (15 to 41)	0.30	20 (13 to 30)	23 (14 to 37)	0.42
Follow–up ^a^	25 (16 to 33)	26 (15 to 43)	0.40	21 (14 to 31)	23 (15 to 41)	0.52
Difference ^b^	1 (0 to 4)	1 (−1 to 3)	0.34	0 (−1 to 2)	0 (−2 to 2)	0.42

ZSC, sodium zirconium cyclosilicate; CPS, calcium polystyrene sulfonate; eGFR, estimated glomerular filtration rate.

Missing data: Sodium minus chloride, 6.8%; Corrected calcium, 28.0%; Phosphorus, 30.3%; Urea nitrogen, 1.5%.

a P value was obtained using the unpaired Student's t-test.

b P value was obtained using the Mann–Whitney U-test.

### Effects on other electrolyte levels and kidney function

Table 2 summarizes the changes in serum levels of sodium minus chloride, corrected calcium, phosphorus, creatinine and urea nitrogen, and eGFR. After propensity score matching, no significant differences were observed in any of the baseline values between the two groups. Serum sodium minus chloride level, a surrogate marker for metabolic acidosis, was significantly elevated in the ZSC group compared with the CPS group (1.9 vs. 1.0 mEq/l, *P* < 0.05), indicating that metabolic acidosis could have been more potentially improved in the ZSC group. Additionally, the CPS group showed slightly higher elevation in serum corrected calcium level (0.1 vs. 0.0 mg/dl, *P* < 0.05), probably due to its calcium content. No significant differences were observed regarding changes in serum phosphorus levels and all markers of kidney function. Similar results were obtained in the linear mixed effects model analysis (Figures 2B–G; Supplementary Table S1).

### Medication retention rate and reasons for discontinuation

Thirteen of 48 patients (27.1%) in the ZSC group and 19 of 84 patients (22.6%) in the CPS group discontinued each potassium binder after the follow-up point, with no significant differences (*P* = 0.67) (Table 3). The most common reason for ZSC cessation was unknown (*n* = 7), followed by hypokalemia (*n* = 3), unpleasant taste (*n* = 2), and other adverse events (*n* = 1). The reasons for CPS cessation were unknown (n = 7), constipation (*n* = 5), hypokalemia (*n* = 2), unpleasant taste (*n* = 2), ineffectiveness (*n* = 2), and other adverse events (*n* = 1). Unknown was the most common reason for discontinuation in both groups, and none of the patients in the ZSC group discontinued treatment due to constipation which was the second common reason in the CPS group.

**Table 3:** Medication persistence according to the type of potassium binders.

**Outcomes**	**ZSC group (*n =* 48)**	**CPS group (*n =* 84)**	***P* value**
**Continuous administration**			
Yes	35 (72.9)	65 (77.4)	0.67
No	13 (27.1)	19 (22.6)	
**Reasons for discontinuation**			
Constipation	0 (0.0)	5 (6.0)	0.16
Unpleasant taste	2 (4.2)	2 (2.4)	0.62
Hypokalemia	3 (6.3)	2 (2.4)	0.35
Ineffectiveness	0 (0.0)	2 (2.4)	0.53
Other adverse events	1 (2.1)	1 (1.2)	1.00
Unknown	7 (14.6)	7 (8.3)	0.38

SC, sodium zirconium cyclosilicate; CPS, calcium polystyrene sulfonate.

## Discussion

Huda et al. have recently reported that ZSC and CPS were equally effective in reducing K^+^ level in patients with hyperkalemia who were admitted to the hospital in an emergency, with comparable cost differences between the two groups (26). However, the differences between these two agents in potassium control and cost after an acute phase including outpatient management remain unclear. To the best of our knowledge, this is the first study to evaluate the difference in the post-acute phase K^+^-lowering effect between ZSC and CPS in patients with non-dialysis dependent CKD. Propensity score methods and linear mixed effect models demonstrated that ZSC controlled K^+^ levels more effectively than did CPS. In addition, a greater increase in sodium minus chloride level, which is only a surrogate indicator for metabolic acidosis, was observed in the ZSC group. These results indicated that ZSC could be a promising therapeutic agent for treatment and prevention of hyperkalemia in patients with CKD.

Patients with CKD are susceptible to developing hyperkalemia due to reduced functioning nephrons, use of RAS inhibitors, concomitant heart failure, and diabetes mellitus (1, 7–9, 27). The prevalence of hyperkalemia increases as kidney function declines, with a rate of 14.6% in patients with non-dialysis dependent CKD and 33.3% in those with end-stage kidney disease (1). The mechanism by which hyperkalemia is associated with high mortality could be attributed not only to life-threatening cardiac arrhythmias by hyperkalemia itself, but also to RAS inhibitor down-titration or discontinuation as a result of hyperkalemia (14, 15). In patients with CKD, restriction of potassium intake is often recommended for managing K^+^ levels. However, several studies have demonstrated that potassium-rich diets including fresh vegetables and fruits were associated with delayed progression of CKD as well as cardiovascular health problems. This beneficial effect might be attributed to other mineral elements, vitamins, and dietary fibers of a high-potassium diet (10, 28, 29). Although there is a lack of evidence for potassium binders to directly provide reno- and cardio-protective effects, these drugs are likely to be useful for maintaining appropriate RAS inhibitor therapy and avoiding excessive restriction of potassium intake. Particularly, RAS inhibitors are critical for preventing progression of diabetic kidney disease (DKD) (30, 31). Moreover, a recent study has demonstrated that a combination of finerenone, a novel MR blocker, and RAS inhibitors could act synergistically to reduce the risk of DKD progression and cardiovascular events in patients with diabetes (32). Given that diabetes is the underlying disease for type 4 renal tubular acidosis with hyperkalemia, RAS inhibitors and MR blockers plus potassium binders could become the standard therapy in patients with DKD in the future.

Although K^+^-lowering effects of ZSC in the present study appeared to be generally equivalent or superior to those in clinical trials, CPS showed only weaker effects than expected, possibly for two reasons (18, 33). First, the lower dose of CPS in the present study compared with the randomized control study (RCT) (15 g per day) may be partially responsible for the modest efficacy of CPS (33). The dose of potassium binders in real-world practice has been reported to tend to be lower probably due to concerns about the gastrointestinal side effects of CPS including constipation, colonic necrosis, and intestinal perforation (12, 13, 34). Second, the potential poor adherence to CPS might have also contributed to the reduction in its efficacy (6). The unpleasant taste and recommended twice-daily or thrice-daily treatment regimens could have a negative impact on patient compliance (33, 35). These clinical shortcomings of CPS could lead to the results of the present study, showing that ZSC was more effective in improving hyperkalemia.

CKD is associated with the development of metabolic acidosis due to impaired ${\text{NH}}_{4}^{+}$ excretion or reduced tubular bicarbonate reabsorption (36). The increase in sodium minus chloride difference was also significant in the ZSC group compared with the CPS group, indicating that ZSC could have the potential to improve metabolic acidosis. Although the mechanism for improved metabolic acidosis with ZSC is poorly understood, it may be due to the effects of direct binding and removal of NH${\text{NH}}_{4}^{+}$ from the gastrointestinal tract and/or increased renal ammoniagenesis with correction of hyperkalemia (37). Since metabolic acidosis is a risk factor for an accelerated decline in kidney function, cardiovascular events, and impaired physical function, patients with advanced CKD often receive sodium bicarbonate supplements (36). Each 5 g dose of ZSC contains approximately 400 mg of sodium, and mild-to-moderate edema has been reported as an adverse event (38). However, considering that ZSC treatment could improve metabolic acidosis and possibly reduce the requirement for sodium bicarbonate, the concern regarding sodium loading by ZSC might be counterbalanced.

A large-scale retrospective study has reported a low continuation rate of conventional potassium binders: approximately 60% of patients with hyperkalemia who had received potassium binders discontinued them for some reason within 1 year (6). It has also been reported that only 10% of new sodium polystyrene sulfonate users continued therapy for more than 60 days (39). Regarding this, we hypothesized that the potentially improved tolerability of ZSC could have facilitated its continuous use (17). However, the medication retention rate beyond the follow-up period was comparable between the ZSC and CPS groups. Although the details were unclear because more than half of the reasons for ZSC discontinuation were not described in the medical record, the high medication cost of ZSC, about eight times that of CPS, might have contributed to its cessation. Kim et al. reported that the cost of prescribing ZSC could be almost fully compensated by savings accompanying the reduced risk of hyperkalemia events requiring hospitalization (40). However, considering that ZSC is less prevalent than anticipated, it is desirable that its drug price be more accessible to many patients.

The present study has several limitations. First, since this was not an RCT but a retrospective observational study, our results should be interpreted with caution. The type of potassium binder was not blinded, which might have led to information bias. Although propensity score matching model and linear mixed effect model analyses were performed to adjust for covariates clinically associated with response to potassium binder and selection of its type, the impact of potential confounders could not be completely ruled out. Second, the nature of this single-center study may have caused selection bias, limiting generalizability. Third, metabolic acidosis was only indirectly assessed using a surrogate marker of serum sodium minus chloride level. Therefore, the results should be interpreted with caution. Additionally, the short follow-up period and small sample size did not allow for adequate evaluation of the continuation rate of ZSC and its adverse events. The above issues should be addressed by robust evidence from future multicenter prospective studies. However, we suggest that the real-world findings of this observational study might be useful for managing hyperkalemia, in which medication and diet adherence play an important role.

In summary, our study demonstrated that ZSC could more potentially improve hyperkalemia and possibly metabolic acidosis in patients with CKD than CPS. Future RCTs are encouraged to clarify the effectiveness of ZSC.

## Data availability statement

The original contributions presented in the study are included in the article/Supplementary material, further inquiries can be directed to the corresponding author.

## Ethics statement

The studies involving human participants were reviewed and approved by Keio University School of Medicine Ethics Committee (approval no.: 20221131). Written informed consent for participation was not required for this study in accordance with the national legislation and the institutional requirements.

## Author contributions

TN and SY contributed to design of the study, collection of the data, and preparation of the initial draft. KH, KU, TT, TA, KM, TY, JY, and TK contributed to interpretation of the data and revision of the manuscript. TM and HI supervised the manuscript. All authors approved the final version of the manuscript.
